# Beating Heart Coronary Artery Bypass Grafting with Preemptive Impella^™^ 5.5 Assist Device in Ischemic Cardiomyopathy

**DOI:** 10.3390/biomedicines13051259

**Published:** 2025-05-21

**Authors:** Francesco Cabrucci, Massimo Baudo, Yoshiyuki Yamashita, Amanda Yakobitis, Courtney Murray, Gianluca Torregrossa

**Affiliations:** 1 Department of Cardiac Surgery Research, Lankenau Institute for Medical Research, Wynnewood, PA 19096, USA; massimo.baudo@icloud.com (M.B.); yamashitay@mlhs.org (Y.Y.); gianluca.torregrossa@gmail.com (G.T.); 2 Department of Cardiac Surgery, Lankenau Medical Center, Wynnewood, PA 19096, USA; yakobitisa@gmail.com (A.Y.); murraycou@mlhs.org (C.M.)

**Keywords:** CABG, Impella 5.5, axial flow pump, heart failure, HFrEF, ischemic cardiomyopathy, beating heart, complex myocardial revascularization

## Abstract

**Background:** Choosing the best surgical approach for coronary revascularization in patients with ischemic cardiomyopathy and low EF is complex. Several strategies have been adopted, including on- and off-pump CABG, the use of IABP, and the combination of ECMO or even LVAD with CABG. Recently, the Impella 5.5 micro-axial pump has been used as perioperative temporary left ventricular support in CABG patients. This study aims to report a series of CABG procedures performed with Impella assistance, highlighting its potential benefits in high-risk surgery cases. **Methods:** Between January 2023 and December 2024, seven consecutive patients underwent on-pump beating CABG with planned central Impella 5.5 support via a 10 mm graft in the ascending aorta. This study focused on assessing perioperative outcomes in patients with reduced ventricular dysfunction (ejection fraction [EF] < 35%) undergoing CABG with Impella-assisted support. **Results:** Seven patients were included in the study, with a median age of 70 [IQR 57–74.7], and six were male. Hypertension was present in all patients, diabetes in six, and COPD in two, and two were in dialysis. The median preoperative EF was 20% [IQR, 18–29%], and the median STS PROM was 5.5 [IQR: 2.9–8.9]. One patient had preoperative IABP support. Four patients required intraoperative transfusions, but all remained hemodynamically stable upon OR exit. The Impella was removed after an average of 5.6 ± 2.1 days. One patient underwent surgical revision for bleeding. No strokes, myocardial infarctions, repeat revascularizations, or mortality occurred postoperatively. The median postoperative hospital stay was 21 [IQR, 17.5–22] days, with a discharge EF of 38% [IQR 33.5–38%]. One patient died 6 months after the procedure due to sepsis caused by a gangrenous diabetic leg. **Conclusions:** This initial experience using Impella 5.5 support in CABG patients with reduced EF demonstrated its feasibility in selected cases. The Impella provided effective circulatory support, ensuring stable hemodynamics throughout the postoperative stay without complications.

## 1. Introduction

Ischemic cardiomyopathy (ICM) is a leading cause of heart failure (HF), with a rising global prevalence and over 8 million cases projected in the U.S. alone by 2030 [1,2]. The management of ICM encompasses a broad spectrum of approaches, including optimal medical therapy, percutaneous and transcatheter interventions, coronary artery bypass grafting (CABG), and advanced HF therapies [3]. Although high-quality evidence remains limited, the cumulative data support CABG as the treatment of choice for patients with ICM, showing superior outcomes compared to medical therapy alone or in combination with Percutaneous Coronary Intervention (PCI) when the surgical risk–benefit balance is favorable [4,5,6].

Surgical myocardial revascularization in ICM is a highly complex operation, and the optimal strategy to achieve it continues to be debated [3]. Beyond the technical aspects of CABG in severely reduced ejection fraction (EF), the surgeons’ challenge in operating these patients is the development of post-cardiotomy cardiogenic shock (PCCS).

PCCS is a dreaded complication that frequently requires high inotropic and vasopressor infusion, often in combination with mechanical circulatory support (MCS) [7,8]. In this setting, short-term MCS is achieved using an intra-aortic balloon pump (IABP), extracorporeal membrane oxygenation (ECMO), a direct left ventricular unloading system with micro-axial flow pumps (Impella^™^, Abiomed Inc., Danvers, MA, USA), or a combination of these modalities (ECMELLA/ECPELLA) [3].

Traditionally, MCS is used as a bailout option in the early postoperative period to counteract PCCS, resulting in high morbidity and mortality [7,9,10]. However, emerging evidence and logic support the prophylactic/preemptive use of MCS in selected high-risk cases [11,12,13]. Impella, with its several configurations and devices, has proven to be a strategic device for counteracting PCCS [14].

We aim to report our early experience with the preemptive use of Impella 5.5 during on-pump beating heart CABG in patients with ICM and severely reduced EF.

## 2. Rationale Behind Our Approach and Selection Criteria

The Impella 5.5 with SmartAssist™ (21 Fr cannula, 19 Fr motor housing, and 9 Fr catheter shaft) is a temporary MCS device designed for short-term use (up to 14 days). It is indicated for patients experiencing ongoing cardiogenic shock within 48 h following an acute myocardial infarction or open-heart surgery when caused by isolated left ventricular failure unresponsive to optimal medical therapy. The primary goal of Impella 5.5 is to directly unload the left ventricle while providing up to 6.2 L/min of continuous blood flow, ensuring adequate systemic perfusion, thereby promoting myocardial recovery [15].

Preemptive left ventricular unloading in ICM patients undergoing CABG aims to (1) preserve end-organ perfusion, (2) avoid the need for high-dose inotropic and vasopressor supports and their side effects, and (3) support ventricular recovery while giving the body more time to counteract the inflammatory response triggered by cardiopulmonary bypass (CPB). On the other hand, preemptive use of the Impella device may have potential drawbacks, including aortic valve injury, bleeding, thromboembolic events, ventricular arrythmias, stroke, limb ischemia, vascular injury, and hemolysis. As a result, the overall balance between risks and benefits remains on the shoulders of the team who pursue the surgery [16].

While awaiting the results of the IMPACT trial (NCT05529654) to shed more light on this ongoing debate, surgeons must individualize treatment by carefully assessing patient-specific factors that may contribute to the development of PCCS [3,17]. In other words, not all patients with ICM requiring CABG are potential candidates for preemptive MCS. A severely dilated ventricle, right ventricular dysfunction, moderate-to-severe tricuspid regurgitation, ventricular dysfunction disproportionate to the ischemic burden, unstable angina, and poor coronary targets are among the key factors that raise suspicion for complicated weaning from CPB and the potential development of PCCS [3]. Moreover, conditions such as severe chronic kidney disease—particularly when associated with challenges in extracellular volume management—as well as social factors that may coexist with or contribute to the development of ICM, should be carefully evaluated [18].

Finally, although our team has successfully adopted off-pump as the primary surgical strategy for all patients requiring surgical revascularization, in this subset of patients with severely dilated LV (LVEDd greater than 6 cm) and very low EF (EF < 30%), on-pump beating heart CABG is our preferred strategy.

In patients with ischemic cardiomyopathy and severely reduced EF, the on-pump beating heart approach offers several potential advantages. It avoids the global myocardial ischemia associated with aortic cross-clamping and cardioplegic arrest, thus reducing the metabolic insult to an already compromised myocardium. The maintenance of native coronary perfusion, particularly in hibernating or stunned myocardium, may facilitate intraoperative hemodynamic stability and enhance early myocardial recovery. Furthermore, avoiding aortic cross-clamping in this fragile cohort may mitigate the risk of embolic stroke and systemic inflammatory response.

However, the role of on-pump beating heart CABG remains controversial [19,20]. A meta-analysis led by Kowalewski et al. raised concerns regarding its routine use [19]. The analysis suggested that, while theoretically beneficial, on-pump beating heart surgery did not consistently translate into improved outcomes when compared to conventional arrested-heart CABG in randomized clinical trials. Critics of the approach argue that suboptimal visualization and anastomotic quality in certain territories, particularly on a beating heart, may offset its hemodynamic advantages. Yet, these concerns must be balanced against the real-world challenges of operating on patients with profound LV dysfunction, in whom myocardial protection strategies must be carefully tailored. In this context, we believe that on-pump beating heart CABG—when combined with contemporary mechanical support such as the Impella 5.5—offers a pragmatic and safe revascularization strategy in high-risk patients with ischemic cardiomyopathy.

## 3. Methods

A retrospective analysis of prospectively collected data was conducted on 96 patients with EF < 40% who underwent CABG at our institution between January 2023 and December 2024. The analysis resulted in 7 patients affected by ICM who underwent on-pump beating heart CABG with preemptive Impella 5.5. All patients, except one, underwent a thorough evaluation by a multidisciplinary heart failure team consisting of cardiologists, anesthesiologists, cardiac surgeons, and a specialized radiologist. Six of seven patients underwent myocardial viability assessment using late gadolinium enhancement magnetic resonance imaging (LGE-MRI), 18F-fluorodeoxyglucose positron emission tomography (18F-FDG PET), or both. The one patient who did not undergo viability assessment presented in cardiogenic shock with significant hemodynamic instability. Left ventricular EF was assessed using transthoracic echocardiography (TTE) and transesophageal echocardiography (TEE) calculated with the Simpson biplane method, following current guideline recommendations.

### Statistical Analysis

Given the limited sample size, assessment of normality was considered unreliable; therefore, non-parametric methods were used, with continuous variables reported as median and interquartile range, and categorical variables presented as frequencies and percentages. Statistical analyses were conducted in R and RStudio (version: 2024.12.0 + 467) employing the “TableOne” package.

## 4. Surgical Technique

The revascularization strategies and preferences of our team have been described elsewhere [21,22]. All patients underwent CABG via full median sternotomy. Following conduit harvesting, CPB was established using standard aortic and right atrial cannulation. CABG was performed on a CPB-assisted beating heart with a pulmonary artery vent in place for the entire grafting procedure. Despite being an indirect venting of the left heart chambers, this solution allowed a full manipulation of the heart during grafting without the risk of left ventricle (LV) free wall injury. Additionally, it provided critical adjunct protection for both the right ventricle (RV) and the LV, especially in the context of severely depressed function.

**Figure 1:**
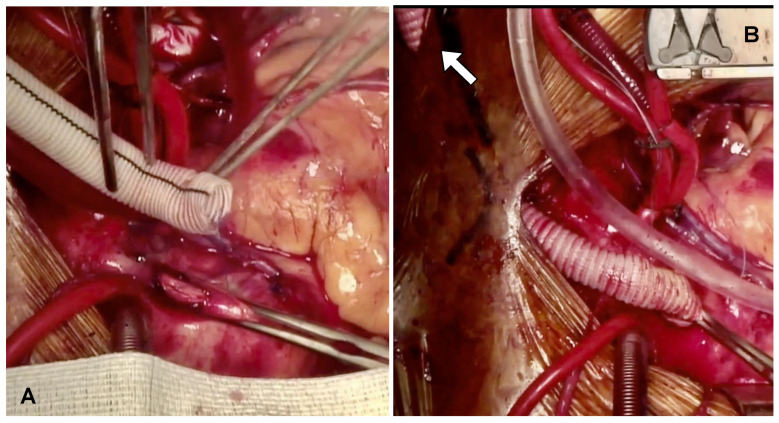
(**A**) A 10 mm Dacron graft is anastomosed to the ascending aorta in an end-to-side fashion. (**B**) The graft is deaired and tunneled to the right supraclavicular region (white arrow), prepared for Impella insertion.

Following completion of grafting, the ascending aorta was side-bite clamped, and a 60-degree beveled 10 mm Dacron graft was anastomosed to the ascending aorta in an end-to-side fashion using a 4/0 Prolene RB1 needle (Ethicon). The Dacron graft was then deaired, and hemostasis was checked. The tube graft was tunneled through the skin of either the right or left supraclavicular region, based on the prefabricated curvature of the Impella device (Figure 1A,B).

**Figure 2:**
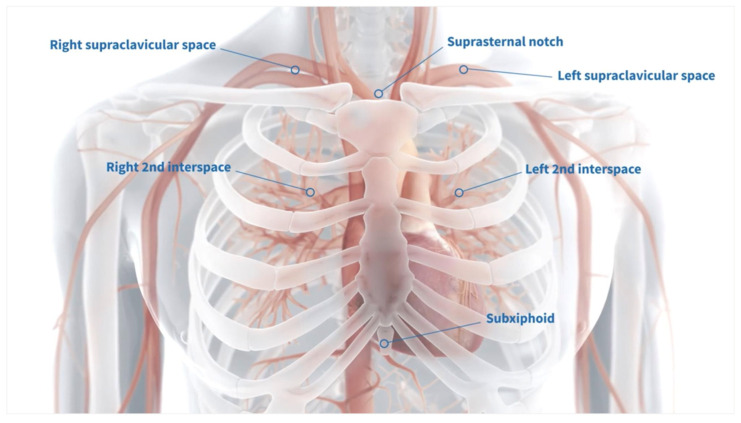
Impella 5.5 externalization options for a direct central aortic approach (adapted with permission from https://www.abiomed.com/en-us/products-and-services/impella/impella-55-with-smartassist Abiomed Inc., Danvers, MA, USA).

Several configurations are available for the externalization of the centrally placed Impella; however, we prefer the supraclavicular region due to its anatomical accessibility and the relative ease of maintaining a clean and manageable exit site (Figure 2).

The micro-axial flow pump Impella 5.5 was inserted using a standardized technique to ensure optimal positioning and minimize complications. After the graft was meticulously deaired, the 23 Fr temporary peel-away sheath included in the Impella insertion kit was inserted and secured using graft locks to maintain stability.

Through this sheath, a 4 Fr diagnostic catheter was introduced—often with the aid of a J wire—to facilitate crossing of the aortic valve. The pigtail catheter was then carefully advanced under TEE guidance into the LV. Once confirmed to be in the correct position, the 0.018” Impella-specific guidewire was advanced into the apex of the LV. The diagnostic catheter was subsequently removed, and a vascular clamp was placed at the base of the graft, proximal to the graft anastomosis, to ensure hemostasis during device insertion.

To prepare the hemostatic valve for passage of the Impella catheter, an 8 Fr coaxial dilator was repeatedly advanced through the peel-away sheath over the 0.018” wire. This step lubricates the valve and facilitates the atraumatic passage of the Impella catheter. The Impella 5.5 device was then loaded onto the wire and advanced through the hemostatic valve and fully into the Dacron graft. Following this, the vascular clamp was released, and the device was smoothly guided through the graft into the ascending aorta and across the aortic valve into the LV cavity.

TEE guidance and close coordination with the anesthesia team were essential throughout this process to confirm appropriate positioning and to avoid complications such as deep LV insertion, which could lead to arrhythmia or free wall injury.

While weaning from CPB, Impella flow was progressively increased to match the patient’s cardiac output requirements and to avoid the use of inotropic or vasopressor agents. Systemic heparinization was then reversed. Meticulous hemostasis was achieved, and the chest was closed in the standard fashion. The patient was then carefully transferred to the intensive care unit (ICU) bed. At this stage, we positioned the patient in a 30-degree head-up position to promote respiratory comfort and airway safety during the early postoperative period. The TEE probe remained in place throughout, and final Impella positioning was performed only after chest closure and bed transfer. Before leaving the operating room (OR), we confirmed that the Impella tip was directed toward the apex of the LV, away from the interventricular septum and mitral valve apparatus, and remained consistently at 3 to 4 cm distal to the aortic valve. This standardized protocol has enhanced procedural efficiency, reduced device-related complications, and ensured stable support in the immediate postoperative period.

In the ICU, patients were maintained in a semi-recumbent position to prevent traction or displacement of the assist device. Extubation was performed per our institutional ICU protocol. Once hemodynamic stability was achieved and a successful weaning trial had been completed, patients were returned to the OR for device explant. The weaning trial consisted of 2 h of support in the P2 range of Impella cardiac power output (CPO). Upon the previously mentioned parameters, Impella was removed, and the Dacron graft was carefully ligated and sutured, and then buried within the subcutaneous tissue. The decision to perform this procedure in the OR was made to ensure maximal sterility and to minimize the risk of surgical site infection.

## 5. Results

Seven patients (six males, median age of 70 [IQR, 57–74.7] years) were included in this study (Table 1). Hypertension and dyslipidemia were present in all patients. Diabetes mellitus and prior myocardial infarction (MI) were observed in six (84%) and five (72%) patients, respectively. Three patients (43%) had preoperative atrial fibrillation (AF), and two (28%) were on dialysis. At the time of referral, five patients (72%) presented in New York Heart Association (NYHA) functional class III or IV, and one was in cardiogenic shock requiring intra-aortic balloon pump (IABP) support. The median preoperative ejection fraction EF was 20% [IQR, 18–29%], and the median Society of Thoracic Surgeons Predicted Risk of Mortality (STS-PROM) score was 5.5% [IQR, 2.9–8.9%]. Four patients (57%) underwent surgery on an elective basis. Intraoperatively, multi-arterial and total arterial revascularization were performed in three (43%) and one (14%) patients, respectively. Concomitant left atrial appendage (LAA) closure was performed in three patients (43%), while two patients (28%) underwent left atrial “box lesion” ablation using the EnCompass™ Clamp (AtriCure, Inc., Mason, OH, USA) [23]. Table 2 shows all demographic and intraoperative variables.

The median total ventilation time was 76 h [IQR: 31–142]. Impella support was weaned after a median duration of 5.6 ± 2.1 days, and no patients developed PCCS. One patient required surgical revision for bleeding. No postoperative strokes, MIs, repeat revascularizations, or in-hospital mortality occurred. The median postoperative hospital stay was 21 [IQR, 17.5–22] days, and the median discharge EF improved to 38% [IQR, 33.5–38%] One patient died six months after surgery due to sepsis secondary to a gangrenous diabetic lower limb. Table 3 summarizes the postoperative outcomes.

## 6. Technical Challenges and Troubleshooting

Centrally placed Impella-supported CABG in the setting of ICM presents a complex clinical and technical landscape. In our experience, several specific intraoperative challenges have been identified, and targeted troubleshooting strategies have been developed accordingly. These aspects are detailed in the following sections.

(1) Minimizing aortic manipulation is critical to reduce the risk of embolic stroke and to preserve the proximal aortic space for secure Impella graft placement. To this end, our preferred strategy involves creating composite grafts based on the in situ internal thoracic arteries (ITAs). We commonly employ a Y-graft from the left internal thoracic artery (LITA), or a bilateral ITA (BITA) configuration, which avoids the need for proximal aortic anastomoses altogether. This approach not only minimizes aortic handling but also preserves valuable “real estate” on the ascending aorta to accommodate the 10 mm Dacron graft required for Impella insertion.
(2) An additional benefit of using ITA-based composite grafting is the ability to employ a “proximal-first” strategy. In this technique, the Y- or I-graft extension (off the LITA or right ITA, respectively) is constructed before the distal anastomoses, enabling immediate reperfusion of distal myocardial territories even before completion of the full revascularization. This is particularly advantageous in the setting of a compromised ventricle, where early restoration of flow may contribute to myocardial protection and improve intraoperative hemodynamics.
(3) When a proximal anastomosis to the aorta is required, we adopt one of two approaches. In some cases, a single side-biting clamp is placed to facilitate both the proximal anastomosis and the Impella graft implantation at the same aortic site, thus limiting manipulation to one location. Alternatively, we utilize a clampless device—such as the Enclose II system (Peters Surgical)—to perform a proximal anastomosis without any aortic clamping, thereby reducing the stroke risk in high-risk patients. When two conduits must be connected to the aorta, we often use the “piggyback” proximal anastomosis technique, in which a single hole is made in the aorta and the second conduit (typically a radial artery or a saphenous vein graft) is anastomosed end-to-side to the hood of the first proximal graft. This technique not only conserves aortic surface area but also limits total aortic manipulation, aligning with our overarching principle of stroke risk reduction and optimized conduit strategy in these vulnerable patients [22].
(4) As soon as the Impella crosses the aortic valve, a degree of aortic regurgitation is typically observed, which can cause LV distension. To mitigate this, the Impella device is promptly activated at performance level P2 CPO, ensuring immediate left ventricular unloading and hemodynamic stabilization.
(5) While most of the literature describes optimal positioning of the Impella 5.5 as 4 to 5 cm beyond the aortic valve into the left ventricle, our institutional experience has demonstrated that a slightly more conservative positioning—approximately 3 to 4 cm—offers sufficient hemodynamic support while minimizing the risk of ventricular wall contact or entanglement with subvalvular structures. A critical aspect of successful positioning is avoiding excessive manipulation of the device during cardiopulmonary bypass (CPB) weaning and surgical hemostasis. In our practice, once the Impella is advanced to a depth of 4 cm into the LV, we proceed with CPB weaning.

## 7. Discussion

In this study, the Impella 5.5 with SmartAssist™ has proven to be a strategic asset in enabling safe and complex coronary revascularization in patients with ischemic cardiomyopathy and severely reduced left ventricular function. Several factors contributed to its value in this high-risk cohort.

First, the centrally placed Impella 5.5 achieved 100% successful deployment, providing robust and reliable LV unloading and ensuring adequate end-organ perfusion and hemodynamic stabilization. This preemptive support facilitated improved end-organ perfusion while significantly reducing the need for vasopressors and inotropes, which can exacerbate myocardial oxygen consumption and promote arrhythmias. Only one patient required minimal vasopressor support upon exiting the operating room, and notably, no cases of PCCS were observed. These results underscore the importance of anticipatory rather than reactive MCS in this surgical population.

Second, the combination of on-pump beating heart CABG and preemptive Impella support allowed for more complete and technically demanding revascularization while avoiding cardioplegic arrest. The use of a beating heart strategy, supported by full-flow CPB and LV unloading, enabled a median of three grafts per patient, achieving complete revascularization. The minimized need for vasoactive agents allowed for broader implementation of multi-arterial (43%) and total arterial (14%) revascularization strategies. In the setting of ICM, the use of multi-arterial strategies has been questioned due to concerns about low early flow of arteries with respect to veins and the longer operative time [3]. However, by avoiding cardioplegic arrest and maintaining continuous perfusion, this technique mitigates those risks. This strategy may be especially beneficial in younger patients or those with viable myocardium territories, where complete and durable revascularization translates into long-term survival gains.

Third, although seemingly more invasive at first glance, our approach involving central placement of the Impella 5.5 proved to be reliable and did not require fluoroscopic guidance, which is often challenging to utilize during open cardiac surgery. In addition, graft interposition into the ascending aorta preserved the axillary artery for future interventions and avoided the risk of thrombus formation or potential competition with internal mammary artery flow. This is particularly relevant when BITA- or LITA-based grafting strategies are employed, where any reduction in subclavian or axillary flow could compromise graft patency. Fourth, the design improvements of the Impella 5.5 over its predecessor (5.0/LD) contributed to procedural safety and ease of use. The device offers better “pushability”—a term used to describe its enhanced shaft stiffness and trackability through the delivery system—and improved handling characteristics. Furthermore, the absence of a pigtail tip reduces the risk of entanglement with the mitral subvalvular apparatus, a previously recognized limitation in small or hypertrophied ventricles.

Our research adds to the expanding literature suggesting that preemptive use of short-term MCS can serve as a viable strategy to facilitate myocardial recovery after high-risk surgical myocardial revascularization in patients with ICM. Our data demonstrated 100% in-hospital survival despite a median STS—PROM of 5.5% (range: 1.92% to 28.9%), with a survival rate of 86% at a median follow-up of [IQR 7.5–16.5] months. These findings are consistent with those reported by Ranganath et al. and the multicenter experience described by Ramzy et al. [11,24]. The potential role of the Impella 5.5 is also being explored in other high-risk surgical contexts, such as isolated mitral valve surgery in ischemic or dilated cardiomyopathy, where it has shown promise in preventing postoperative ventricular failure [12]. In the context of heart failure surgery, these findings are encouraging and support the concept of preemptive use of the Impella 5.5, rather than its deployment as a rescue therapy after PCCS has already developed—a concept further reinforced by the significantly higher mortality associated with delayed intervention once PCCS is established [10,25]. Our findings support the concept of “Impella-protected cardiac surgery”. This forward-thinking approach aims to optimize myocardial preservation, facilitate more complete and durable revascularization, and improve early and mid-term outcomes of a challenging and high-risk patient population. While awaiting the results of the IMPACT trial to draw more definitive guidance, our experience suggests that the preemptive use of the Impella 5.5 in selected high-risk surgical patients is both safe and effective. It may ultimately become a cornerstone strategy in the management of complex ischemic cardiomyopathy [17].

## 8. Limitations

This study is limited by its small sample size and non-comparative design, which restrict the generalizability of the findings. Additionally, the lack of a control group limits the ability to assess outcomes relative to standard care. Furthermore, the inclusion of both urgent and elective cases reflects the real-world clinical setting of this case series. While this introduces some degree of heterogeneity in baseline risk profiles, such variability is inherent to observational reports of high-risk surgical populations. Cost considerations related to the use of the Impella 5.5 device are debated and also warrant further evaluation, particularly in the context of broader clinical adoption and healthcare resource allocation [26,27]. Finally, this study represents the experience of a single center, which may limit the generalizability of the findings to broader populations.

## 9. Conclusions

Preemptive Impella 5.5 support may redefine the surgical management of ischemic cardiomyopathy, offering a safe and effective bridge through high-risk revascularization. Our findings reinforce the shift toward a “protected cardiac surgery” paradigm. Further studies will determine its broader impact on long-term outcomes.

## Figures and Tables

**Table 1:** Summary of the demographics and clinical variables of each single case.

Case	Age, Sex	Preoperative Findings	Heart Failure Status	Target Vessels	Grafts	Procedure	# Days with Impella	Follow-Up Months
**1**	70, M	STS score: 28.98 COPD: No DM: No Dialysis: Yes Surgical Priority: Urgent	Prior MI: STEMI EF: 30% MRI: No PET: No	LAD: 95% OM: CTO PDA: CTO	LITA to LAD, SVG to PDA	On-pump beating CABGx2, LAA Clip, Impella 5.5	4	11, alive
**2**	78, M	STS score: 5.48 COPD: Yes DM: Yes, type 2 Dialysis: No Surgical Priority: Elective	Prior MI: NSTEMI EF: 28% MRI: Yes PET: No	LAD: 95% OM: 80% PDA: 90%	LITA to LAD, LRAD Y-graft from LITA to OM, SVG to PDA	On-pump beating CABGx3, Impella 5.5	6	22, alive
**3**	75, M	STS score: 7.23 COPD: Yes DM: Yes, type 2 Dialysis: No Surgical Priority: Urgent	Prior MI: NSTEMI EF: 13% MRI: Yes PET: No	LMCA: 70% OM: 99% RCA: CTO	LITA to LAD, SVG sequential to OM- PDA, SVG to D1 (proximal piggyback)	On-pump beating CABGx4, LAA Clip, EnCompass Box Lesions, Impella 5.5	4	21, alive
**4**	63, M	STS score: 1.24 COPD: Yes, type 2 DM: No Dialysis: No Surgical Priority: Elective	Prior MI: NSTEMI EF: 18% MRI: No PET: Yes	LAD: 85% OM: CTO PDA: 95%	LITA to LAD, SVG to PDA, SVG to OM (proximal piggyback)	On-pump beating CABGx3, Impella 5.5	5	10, alive
**5**	49, F	STS score: 10.60 COPD: No DM: Yes, type 1 Dialysis: No Surgical Priority: Elective	Prior MI: No EF: 20% MRI: No PET: Yes	LAD: CTO OM: 90% RCA: CTO	LITA to LAD, SVG to PDA, SVG to OM (proximal piggyback), LRAD Y-graft from SVG to RI	On-pump beating CABGx4, Impella 5.5	4	12, alive
**6**	51, M	STS score: 3.81 COPD: No DM: Yes, type 2 Dialysis: Yes Surgical Priority: Urgent	Prior MI: NSTEMI EF: 18% MRI: Yes PET: No	LAD: 85% OM: 99% RCA: CTO	LITA to LAD, RITA I-graft with SVG to PDA	On-pump beating CABGx2, Impella 5.5	10	5, death
**7**	75, M	STS score: 1.92 COPD: No DM: Yes, type 2 Dialysis: No Surgical Priority: Elective	Prior MI: No EF: 28% MRI: Yes PET: Yes	LMCA: 90% OM: 95% PDA: non-dominant	LITA to LAD, LRAD Y-graft from LITA to OM	On-pump beating CABGx2, LAA clip, EnCompass Box Lesions, Impella 5.5	6	5, alive

STS—Society of Thoracic Surgeons; CABG—coronary artery bypass grafting; COPD—chronic obstructive pulmonary disease; DM—diabetes mellitus; MI—myocardial infarction; EF—ejection fraction; MRI—magnetic resonance imaging; PET—positron emission tomography; (N)STEMI—(non) ST segment enhanced myocardial infarction; LAD—left anterior descending artery; OM—obtuse marginal artery; PDA—posterior descending artery; LAA—left atrial appendage; LITA—left internal thoracic artery; RITA—right internal thoracic artery; SVG—saphenous vein graft; LRAD—left radial artery.

**Table 2:** Demographics and intraoperative variables.

	Overall
**(n)**	7
**Age** (median [IQR])	70 [57–74.7]
**Male** (%)	6 (85)
**STS Score** (median [IQR])	5.5 [2.9–8.9]
**BMI** (median [IQR])	26.5 [23.9–33.5]
** Diabetes ** (%)	6 (85)
** Hypertension ** (%)	7 (100)
** Dyslipidemia ** (%)	7 (100)
** Pre-Surgery Dialysis ** (%)	2 (28)
** COPD ** (%)	2 (28)
** NYHA III/IV ** (%)	5 (72)
** Cerebrovascular Disease ** (%)	0 (0)
** Peripheral Vascular Disease ** (%)	2 (28)
** Preop AF ** (%)	3 (43)
** Liver Disease ** (%)	1 (14)
** Mediastinal Radiation ** (%)	1 (14)
** Prior MI ** (%)	5 (72)
** Previous PCI ** (%)	0 (0)
** Cardiogenic Shock ** (%)	1 (14)
**Pre-Surgery EF** (median [IQR])	20 [18–29]
**Right Ventricular Dysfunction** (%)	5 (72)
**Elective Surgery** (%)	4 (57)
**IABP Preop** (%)	1 (14)
**Time in OR** (hours) (median [IQR])	7.5 [7.0–7.9]
**CPB Time** (min) (median [IQR])	141 [125.5–147]
**Left Atrial Appendage Clipping** (%)	3 (43)
**Second Arterial Conduit** (%)	3 (43)
**Total Arterial Revascularization** (%)	1 (14)

STS—Society of Thoracic Surgeons; BMI—body mass index; NYHA—New York Heart Association; COPD—chronic obstructive pulmonary disease; AF—atrial fibrillation; PCI—percutaneous coronary intervention; MI—myocardial infarction; EF—ejection fraction; IABP—intra-aortic ballon pump; OR—operating room; CPB—cardiopulmonary bypass.

**Table 3:** Postoperative outcomes.

	Overall
**(n)**	7
** Re-Exploration for Bleeding ** (%)	1 (14)
** Patients Required Vasopressor at OR Exit ** (%)	1 (14)
** Postoperative Ventilation Time ** (hours) (median [IQR])	76 [31–142]
**Impella Assistance** (days) (median [IQR])	5 [4–6]
** AKI ** (%)	1 (14)
** New Postoperative Dialysis ** (%)	0 (0)
** Postoperative AF ** (%)	2 (28)
**Postoperative Stroke** (%)	0 (0)
** Superficial Infection ** (%)	3 (43)
** Deep Sternal Infection ** (%)	0 (0)
** Unplanned Coronary Intervention ** (%)	0 (0)
**ICU LOS** (days) (median [IQR])	6.2 [4.7–7.6]
** 30 Days of In-Hospital Mortality ** (%)	0 (0)
**Postoperative EF % at Discharge** (median [IQR])	38 [33.5–38]
**Hospital LOS** (days) (median [IQR])	21 [17.5–22]
**Survival at Median Follow-Up** (11 [IQR 7.5–16.5] months) %	86%

AKI—acute kidney injury; AF—atrial fibrillation; ICU—intensive care unit; IQR—interquartile range; LOS—length of stay; EF—ejection fraction; OR—operating room.

## Data Availability

The raw data supporting the conclusions of this article will be made available by the authors upon request and institutional approval.
